# Copper-based metal-organic frameworks (BDC-Cu MOFs) as supporters for α-amylase: Stability, reusability, and antioxidant potential

**DOI:** 10.1016/j.heliyon.2024.e28396

**Published:** 2024-03-19

**Authors:** Sami A. Al-Harbi, Yaaser Q. Almulaiky

**Affiliations:** a Department of Chemistry, University College in Al-Jamoum, Umm Al-Qura University, Makkah, Saudi Arabia; b Department of Chemistry, Collage of Science and Arts at Khulis, University of Jeddah, Jeddah, Saudi Arabia; c Chemistry Department, Faculty of Applied Science, Taiz University, Taiz, Yemen

**Keywords:** α-Amylase, Antioxidant activity, Immobilization, Metal-organic frameworks

## Abstract

Copper-based metal-organic frameworks (BDC-Cu MOFs) were synthesized via a casting approach using 1,4-benzene dicarboxylic (BDC) as organic ligand and their properties characterized. The obtained materials were then utilized to immobilize the α-amylase enzyme. The chemical composition and functional components of the synthesized support (BDC-Cu MOFs) were investigated with Fourier transform infrared spectroscopy (FTIR), the surface morphology was determined with scanning electron microscopy (SEM), and the elemental composition was established with energy dispersive X-ray (EDX) analyses. X-ray diffraction (XRD) was employed to analyze the crystallinity of the synthesized DBC-Cu MOFs. The zeta potentials of DBC-Cu MOFs and DBC-Cu MOFs@α-amylase were determined. The immobilized α-amylase demonstrated improved catalytic activity and reusability compared to the free form. Covalent attachment of the α-amylase to BDC-Cu provided an immobilization yield (IY%) of 81% and an activity yield (AY%) of 89%. The immobilized α-amylase showed high catalytic activity and 81% retention even after ten cycles. Storage at 4 °C for eight weeks resulted in a 78% activity retention rate for DBC-Cu MOFs@α-amylase and 49% retention for the free α-amylase. The optimum activity occurred at 60 °C for the immobilized form, whereas the free form showed optimal activity at 50 °C. The free and immobilized α-amylase demonstrated peak catalytic activities at pH 6.0. The maximum reaction velocities (Vmax) values were 0.61 U/mg of protein for free α-amylase and 0.37 U/mg of protein for BDC-Cu MOFs@α-amylase, while the Michaelis‒Menten affinity constants (Km) value was lower for the immobilized form (5.46 mM) than for the free form (11.67 mM). Treatments of maize flour and finger millet samples with free and immobilized α-amylase resulted in increased total phenolic contents. The enhanced antioxidant activities of the treated samples were demonstrated with decreased IC_50_ values in ABTS and DPPH assays. Overall, immobilization of α-amylase on BDC-Cu MOFs provided improved stability and catalytic activity and enhanced the antioxidant potentials of maize flour and finger millet.

## 1 Introduction

Enzymes are biomolecules that serve as catalysts for biochemical reactions, and they find widespread use across various fields. The broad utilization of these molecules can be attributed to their gentle reaction conditions, specific substrate preferences, and eco-friendliness [1,2]. Enzymes are significant catalysts that play crucial roles in biosensors and other biotechnological processes [[3], [4], [5]]. α-Amylase is one of the most commonly used amylolytic enzymes in the food, textile, detergent, pharmaceutical, paper, and leather industries [[6], [7], [8]]. To release short-chain oligosaccharides from polysaccharides, a glycoside hydrolase (GH) must first break their α-1-4 glucosidic linkages. However, enzymes have limitations, as they tend to lose their original structures when exposed to harsh environmental conditions. To provide an appropriate environment for effective enzyme activity, companies are exploring various alternatives [9]. Immobilization may overcome these limitations. Immobilized enzymes are biocatalysts that are fixed or confined to a support matrix or surface, which provides a stable and reusable enzyme system. Immobilization enhances the stabilities, activities, and selectivities of enzymes while also facilitating separation and recovery of the enzyme after it has catalyzed the substrate reaction. Immobilized enzymes have found extensive use in various industrial applications, such as in the production of food and beverages, pharmaceuticals, and biofuels. Despite the numerous advantages of using immobilized enzymes, there are also certain limitations to this approach. Immobilization sometimes leads to a reduction in enzyme activity or a decrease in the reaction rate due to alterations in the enzyme conformation or its accessibility to the substrate. Furthermore, the immobilization process can be costly and time-consuming, which may restrict usage in certain applications [[10], [11], [12]]. For a material to be suitable as a support, it must retain the maximum possible level of enzyme activity while also protecting the enzyme and permitting reuse in practical applications. A variety of support materials have been developed to protect enzymes, including metal-organic frameworks (MOFs) [[13], [14], [15], [16]]. Self-assembly of rigid organic bridging ligands and metal ions leads to the creation of metal-organic frameworks (MOFs), which are novel organic‒inorganic hybrid nanomaterials [17,18]. These materials are very versatile and can be utilized in a broad range of applications due to their well-organized porous crystal structures, adjustable pore sizes, and ease of chemical modification [19]. They have gained significant attention in recent years due to their unique properties and potential for application in various fields, including drug delivery [20], dye and metal adsorption [21], chromatographic separation [22], catalysis [23], and sensing [24]. MOFs have recently emerged as promising materials for enzyme immobilization [[25], [26], [27]].

MOFs have several unique properties that make them ideal for enzyme immobilization. Their high surface areas and adjustable pore sizes enable effective enzyme loading and provide precise control over enzyme immobilization. Additionally, MOFs can safeguard the enzymes from harsh conditions, such as high temperatures, extreme pHs, and organic solvents, which often lead to enzyme denaturation and activity loss. This is accomplished by confining the enzymes within the MOF pores, which shields them from the external environment. Furthermore, the functional groups within the MOFs facilitate enzyme immobilization through covalent bonding or electrostatic interactions. In recent years, there has been growing interest in using MOFs as support materials for enzyme immobilization [[28], [29], [30]]. Zeyadi et al. [31] reported the immobilization of horseradish peroxidase onto an NH_2_-MOF-Zr through covalent bonding. The resulting biocatalyst exhibited reusability and higher efficacy in removing phenol than the free enzyme. Atiroğlu et al. [32] also reported the immobilization of α-amylase onto OLB/BSA@ZIF-8 MOF with covalent bonding and adsorption. The resulting biocatalyst showed high activities and stabilities at different pHs and temperatures. Acet et al. reported the immobilization of α-amylase onto Carbon felt modified with Ni^2+^ ions. The resultant biocatalyst demonstrated superior reusability and increased efficiency in the degradation of starch compared to the free enzyme [33].

The objective of this study was to immobilize α-amylase on copper integrated with 1,4-benzene dicarboxylic (BDC-Cu MOFs). The support material was characterized using X-ray diffraction (XRD), scanning electron microscopy (SEM), Fourier transform infrared spectroscopy (FTIR), and zeta potential measurements. The immobilization parameters were optimized for high efficiency. Kinetic parameters, temperature, pH, and storage stabilities were evaluated to compare the properties of the immobilized enzyme with soluble α-amylase.

This study has several novel contributions. Firstly, it introduces the synthesis of copper-based metal-organic frameworks (BDC-Cu MOFs) via a casting approach, which is a less explored method. Characterization techniques, including FTIR spectral analysis, SEM, EDX analysis, and XRD, provided a comprehensive understanding of the synthesized BDC-Cu MOFs. Additionally, immobilizing α-amylase on BDC-Cu MOFs represents a novel application of this specific MOF. The evaluation of stability, reusability, and catalytic activity of the immobilized α-amylase, along with its enhanced performance compared to the free form, adds to the novelty of this research. Lastly, the study demonstrates the potential of BDC-Cu MOF-supported α-amylase in enhancing the antioxidant properties of maize flour and finger millet, which is a unique contribution of this specific MOF.

## 2 Results and dissuasion

### 2.1 Synthesis of the material support (BDC-Cu)

**Scheme 1:**
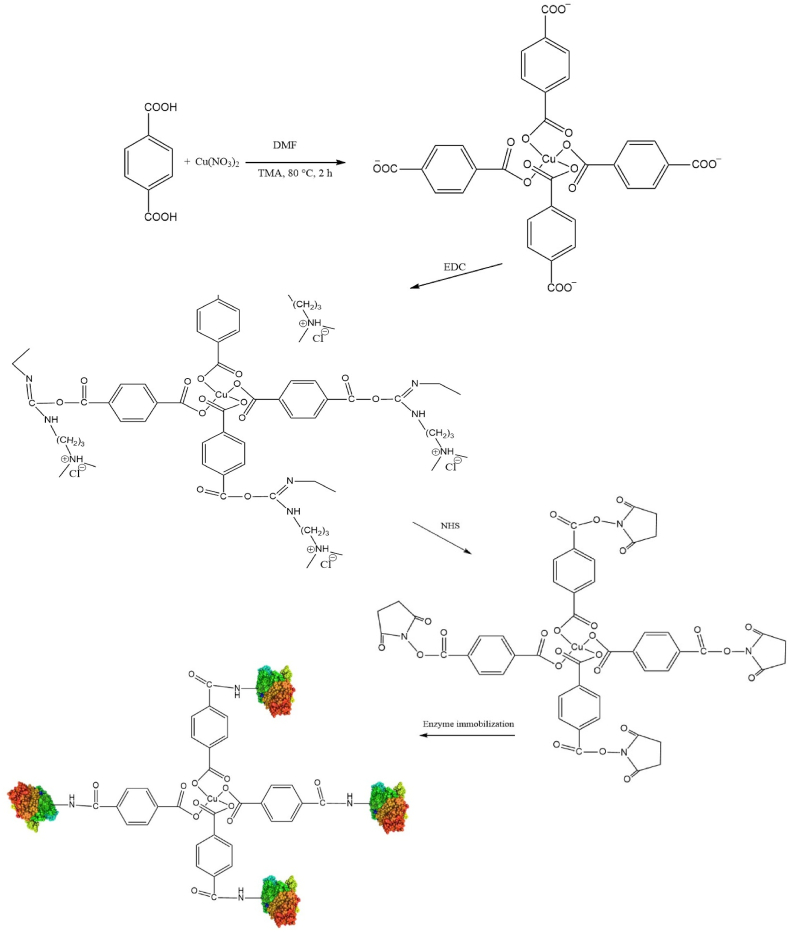
Synthesis of the BDC-Cu MOFs and enzyme immobilization.

The synthetic strategies employed in nanoparticle fabrication are designed to optimize various physicochemical properties, morphologies, and crystallite sizes to achieve improved stabilization, monodispersity, and biocompatibility. In this particular study, a casting approach was chosen for the synthesis of BDC-Cu MOFs due to its effectiveness in controlling the morphologies, uniformities, surface charges of particles, crystallite sizes and agglomeration [34]. Trimethylamine (TMA), an organic base, played a crucial role in the MOF synthesis by deprotonating the organic linker molecules or ligands. This deprotonation process facilitated coordination of the metal ions with the metal-ligand bonds that provide the stability of the MOF structure [35]. In this study, copper-based metal-organic frameworks (MOFs) were synthesized by using 1,4-benzene dicarboxylic (BDC) as the organic linker in the presence of N,N-dimethylformamide, as illustrated in Scheme 1. The resulting coordination complex contains 1,4-benzene dicarboxylic ligands that are coordinated to copper ions (Cu^2+^) in a bidentate bridging fashion. Each Cu^2+^ atom is also coordinated by a molecule of DMF, resulting in a square-pyramidal coordination geometry for the Cu^2+^ atoms. This arrangement provided a specific structure in which the Cu^2+^ atoms were coordinated to the BDC linkers in the (201) planes. These planes or sheets were then connected through weak stacking interactions, which are similar to those observed in a material called MOF-2 [36]. Clausen et al. reported the structure of a polymorph of MOF-2 that shared the same space group and exhibited unit-cell parameters similar to those of the described complex [37]. The resulting MOFs were obtained as solid crystalline materials. The synthesized MOF crystals were subjected to characterization techniques to analyze their properties. Furthermore, the obtained MOF crystals were utilized for immobilizing the enzyme α-amylase. The covalent binding of α-amylase to BDC-Cu resulted in an immobilization yield (IY%) of 81% and an activity yield (AY%) of 89%. These findings were compared to those in previous research, in which immobilization of the HRP enzyme on NH_2_-MOF-Zr exhibited an immobilization yield of 76% and an activity yield of 82% [31]. HRP immobilization on CuONS-PMMA yielded an immobilization yield of 73% [38].

In another study by Cao et al. [39], soybean epoxide hydrolase was immobilized on UIO-66-NH_2_ with an enzyme activity of 88.0%. Salgaonkar et al. [40] reported that glucoamylase and α-amylase enzymes were successfully immobilized in a metal-organic framework (MOF) structure, resulting in a yield of 68% and an enzyme activity of 73.3 units per milligram. Immobilization of the α-amylase enzyme was attributed to the formation of permanent crosslinks between the enzyme and the carrier via EDC/NHS. This hypothesis was supported by strong covalent binding of the enzyme to BDC-Cu, which also exhibited efficient loading and α-amylase performance. The effectiveness of the enzyme was enhanced by reduction of the steric barriers surrounding its active sites, which was facilitated by the large surface area of the carrier and the presumed even distribution of the enzyme [41]. The establishment of robust and distinct chemical linkages within the MOFs and enzymes was primarily influenced by covalent bonding [42]. The presence of multiple covalent bonds on the surfaces of MOFs and enzymes minimizes their structural flexibility and provides stability, preventing protein leakage, unfolding, collapse, or denaturation [43].

## 3 Characterization of the material support

### 3.1 FTIR analysis

**Fig. 1:**
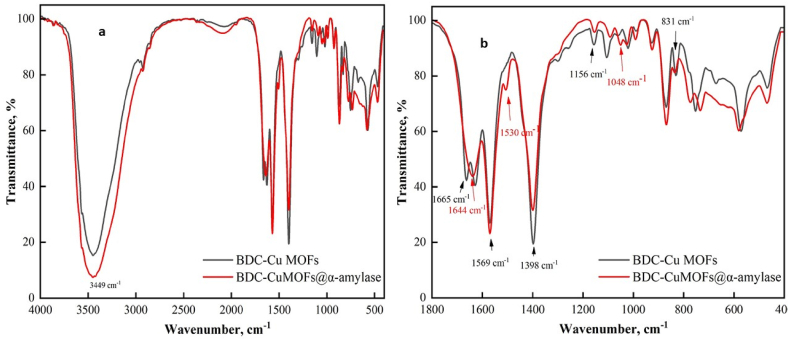
ATR-FTIR spectra of BDC-Cu MOFs before and after enzyme immobilization (a) full and b) expanded scales).

The chemical composition and functional components of the synthesized supporter and BDC-Cu MOFs@α-amylase were investigated with FTIR spectral analyses over the range from to 4000 cm^−1^ (Fig. 1). The FTIR analysis revealed the presence of O–H asymmetric stretching vibrations at 3449 cm^−1^. The bands observed at 1665 cm^−1^ and 1569 cm^−1^ corresponded to the C=O stretching vibrations of the carbonyl group in BDC, as well as the C–C skeletal vibrations of the aromatic ring. The strong band at 1398 cm^−1^ was assigned to C–O stretching vibrations. Moreover, the absorption bands at 831 cm^−1^ and 1156 cm^−1^ were attributed to symmetric and asymmetric stretching vibrations of O–C=O. The presence of immobilized α-amylase enzyme was confirmed by the changes seen in the substrate spectrum following enzyme immobilization. Additionally, the appearance of a glycosidic C–O–C band at 1048 cm^−1^, along with a broad band at 1644 cm^−1^ corresponding to C=O stretching vibration (amide I), and a weak band at 1530 cm^−1^, were attributed to C–N stretching and N–H bending vibrations (amide II).

### 3.2 Morphological characterization of the support

**Fig. 2:**
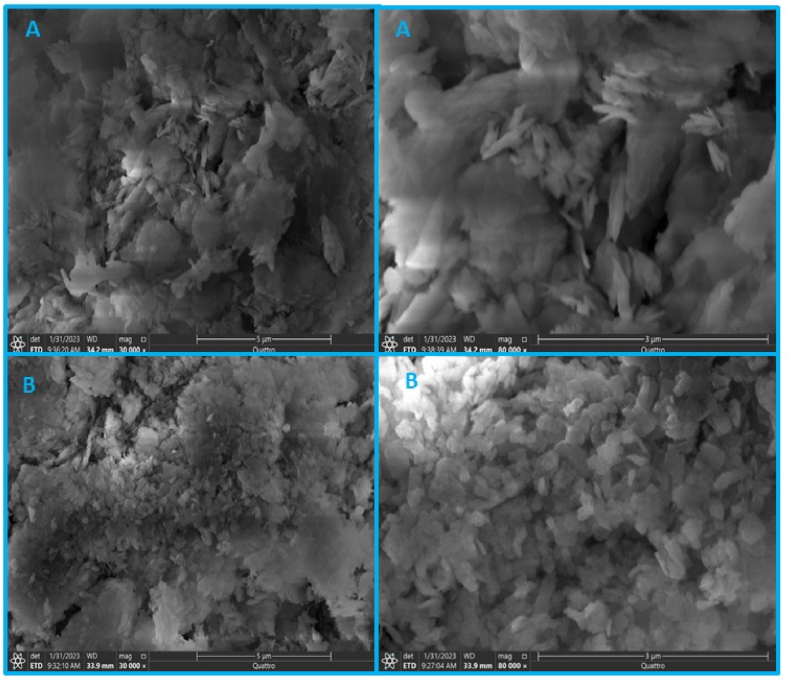
FESEM images of a) BDC-Cu MOFs and b) BDC-Cu MOFs@α-amylase

**Fig. 3:**
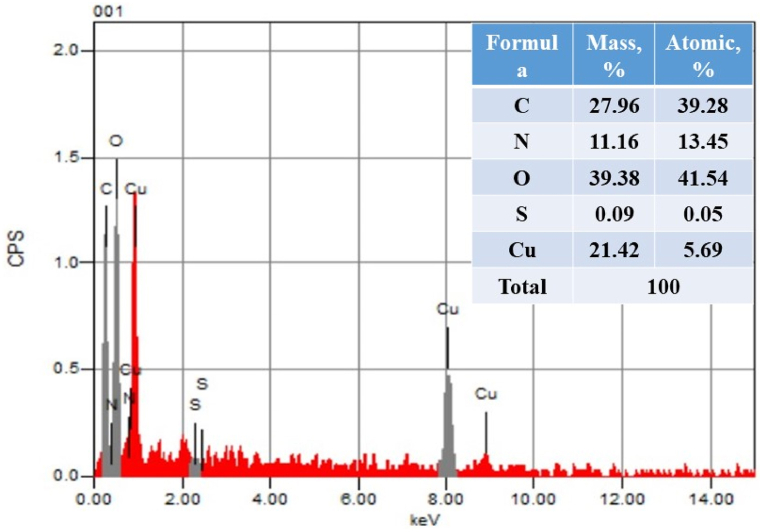
The SEM–energy-dispersive X-ray (EDX) spectra of BDC-Cu MOFs@α-amylase

SEM and EDX analyses were used to study the surface morphologies and elemental compositions of BDC and BDC-Cu MOFs@α-amylase, as shown in Fig. 2. The SEM micrograph in Fig. 2a revealed the distribution of particles in the copper MOF, which appeared as irregularly shaped flakes arranged in wave clusters. The image clearly demonstrated that the particles were highly agglomerated. In Fig. 2b, the SEM image displays the changes occurring in the surface morphology of DBC-Cu MOFs after the immobilization of α-amylase. Compared to the morphology of the DBC-Cu MOFs alone, there were observable changes in the appearance of the surface. These changes included variations in particle sizes, distributions, and overall shapes. The EDX analysis of DBC-Cu MOFs@α-amylase revealed the presence of five elements, namely, copper (Cu, 21.42%), carbon (C, 27.96%), oxygen (O, 39.38%), nitrogen (N, 11.16%), and sulfur (S, 0.09%). This composition suggested the presence of proteins (α-amylase) on the surface of the DBC-Cu MOFs (Fig. 3).

### 3.3 X-ray diffraction analysis

**Fig. 4:**
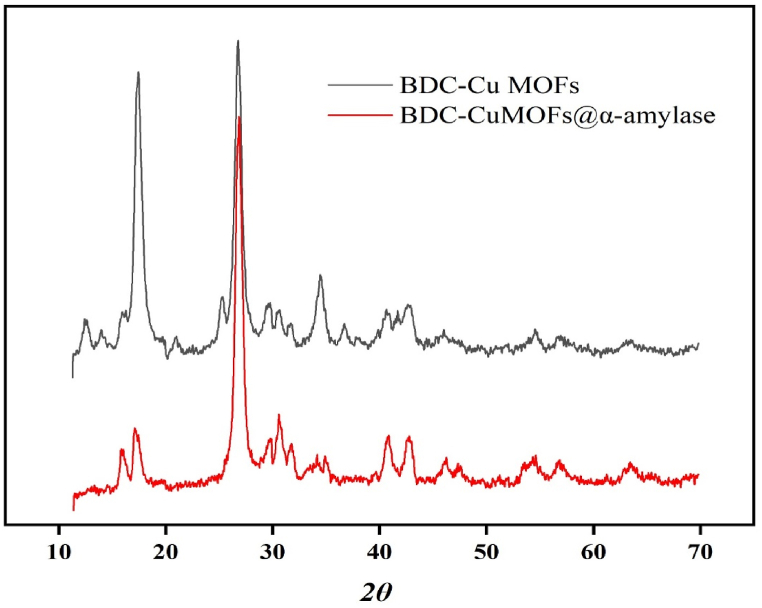
XRD patterns for BDC-Cu MOFs and BDC-Cu MOFs@α-amylase.

X-ray diffraction (XRD) was used to analyze the crystallinities of the synthesized materials. Fig. 4 displays the XRD pattern for the DBC-Cu MOFs. The pattern exhibited prominent high-intensity Bragg diffraction peaks at 2θ values of 17.5° and 26.7°. Additionally, a few low-intensity peaks were observed, indicating the presence of the copper metal-organic framework (MOF) in the sample. After immobilizing the enzyme, the XRD pattern showed that the diffraction peaks at 17.5° and 26.7° remained unchanged, indicating that these specific crystalline structures were unaffected by the immobilization process. However, other diffraction peaks in the XRD pattern were shifted, suggesting alterations in the crystallographic properties of the material due to the interactions between α-amylase and the support material. These interactions included: 1) bonding of the enzyme was facilitated by the presence of carboxylic groups in DBC, and 2) strong immobilization by the DBC-Cu MOFs was attributed to the ability of Cu to facilitate cross-linking of the regular open frame structure of the DBC [44].

### 3.4 Zeta potentials and the average hydrodynamic sizes of particles

The zeta potentials of DBC-Cu MOFs and DBC-Cu MOFs@α-amylase are illustrated in Table 1 and Fig. 1, Fig. 2S (supplementary file). The DBC-Cu MOFs showed a zeta potential of −8.29, which indicated that the DBC-Cu MOF particles had negative surface charges in the liquid medium. This could have arisen from several factors, including the presence of charged functional groups on the surface of the MOFs, dissociation of the ionizable groups, or adsorption of charged species from the surrounding medium. After enzyme immobilization, the surface charge of the material support may undergo changes due to attachment of the enzymes or other biomolecules. This can lead to changes in the zeta potential of the material support. The change in the zeta potential from −8.29 to −10.5 after the immobilization of α-amylase indicated an increased negative surface charge for the DBC-Cu MOF particles. Immobilization of α-amylase onto the DBC-Cu MOFs may have introduced additional charged functional groups or altered the surface properties of the MOFs, resulting in the more negative zeta potential. The α-amylase molecules themselves contain charged residues or groups that contributed to the overall surface charge of the immobilized system [45].

**Table 1:** Zeta potentials and intensity weighted mean hydrodynamic sizes of DBC-Cu MOF and DBC-Cu MOF@α-amylase

Sample	Zeta potential (mV)	Z-Average (d.nm)
DBC-Cu MOFs	−8.29	2145
DBC-Cu MOFs@α-amylase	−10.5	4913

Overall, the change in zeta potential to a more negative value (−10.5) after the immobilization of α-amylase suggested an alteration in the surface charge of the DBC-Cu MOFs due to the presence and interactions of the immobilized α-amylase. The intensity weighted mean hydrodynamic size, also known as the Z-average, is used to describe the hydrodynamic size distribution of particles in a sample. It is important to note that the Z-Average is a measure of the hydrodynamic size, which includes the size of the particle or molecule as well as the surrounding solvent or hydration layer. In this study, the Z-average of the material support before immobilization of the α-amylase was 2145 d nm. This was the average diameter of the particles or molecules comprising the material support. After the immobilization of α-amylase, the Z-average increased to 4913 d nm. This increase in the Z-average suggested that the sizes or hydrodynamic diameters of the particles increased on the material support. This was attributed to the presence of the immobilized α-amylase, which likely contributed to the larger overall size of the system. The increased Z-average could be due to the binding of α-amylase molecules onto the material support, leading to the formation of larger aggregates or complexes [46].

### 3.5 Reusability and stability

**Fig. 5:**
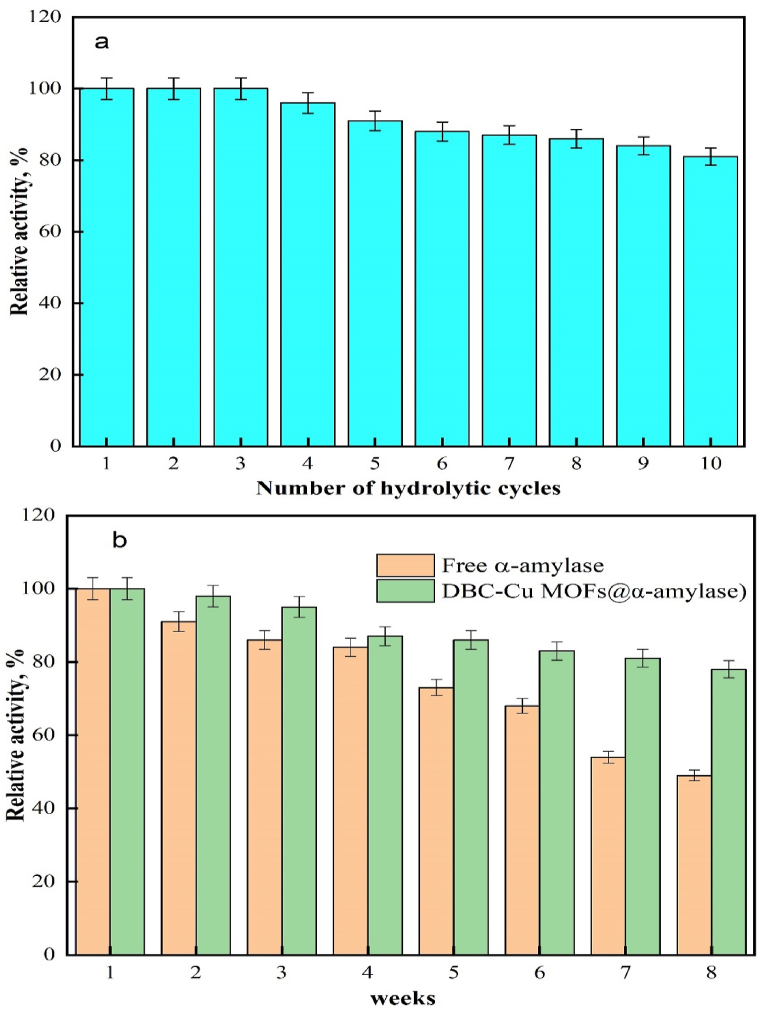
(a) Reusability of immobilized α-amylase and (b) Storage stability of free and immobilized α-amylases.

Enhanced usability, maximized catalyst cycling, and reduced costs were the key objectives for the immobilization of α-amylase. The reusability of the immobilized α-amylase is demonstrated in Fig. 5a, revealing a significant improvement in both the catalytic activity and reusability compared to those of free α-amylase. Unlike free α-amylase, which can only be used once, the immobilized form retained approximately 81% of its catalytic activity even after the 10th cycle. This confirmed the benefit of immobilizing α-amylase, which led to cost reductions and improved economic benefits. Consequently, the immobilized α-amylase exhibited a substantial increase in reusability, laying a solid foundation for future use in biocatalysis. These results support the notion that different carrier supports, such as a modified acrylic fabric [1] and amidoximated acrylic fabric [47], preserved the activity of α-amylase during multiple repetitions. Specifically, the modified acrylic fabric retained 72% of its original activity after 10 repetitions, while the amidoximated acrylic fabric maintained 50% of its original activity after 15 repetitions. The loss of activity seen after repeated cycling could have resulted from alterations in the enzyme structure or adsorption of substrates and products at the reactive sites [13].

To evaluate the storage stabilities of the free and immobilized α-amylases, their relative activities were assessed following incubation in sodium acetate buffer (50 mM, pH 5.5) at 4 °C for 8 weeks. The results demonstrated that the DBC-Cu MOFs@α-amylase and free α-amylase retained 78% and 49% of their initial activities, respectively, after an 8-week storage period at 4 °C (as depicted in Fig. 5b). The immobilized α-amylase exhibited significantly improved storage stability compared to the free enzyme. The DBC-Cu MOFs@α-amylase, in particular, demonstrated superior retention of enzymatic activity, suggesting its potential for prolonged storage and subsequent use in various biocatalytic applications.

These findings were consistent with previous studies conducted by other researchers, who also observed that immobilized enzymes retained their enzymatic activity more effectively than free enzymes during storage. For instance, Dhavale et al. [48] reported that free α-amylase retained only 18% of its activity after 20 days, whereas amylase immobilized on chitosan-coated MNPs preserved 66% of its activity during the same period. Similarly, Sohrabi et al. [49] found that α-amylase immobilized on silica-coated Fe_3_O_4_ nanoparticles maintained up to 79% of its activity after 12 days of storage. These results highlight the increased storage stability conferred by enzyme immobilization. The enhanced storage stability was attributed to the robust and stable structure of the immobilized enzyme on the surface of MOFs (metal-organic frameworks) [50]. This enhanced stability was explained by the immobilization process, which provided a rigid support for the enzyme and protected it from external factors that may cause degradation or denaturation.

### 3.6 Effects of temperature and pH

**Fig. 6:**
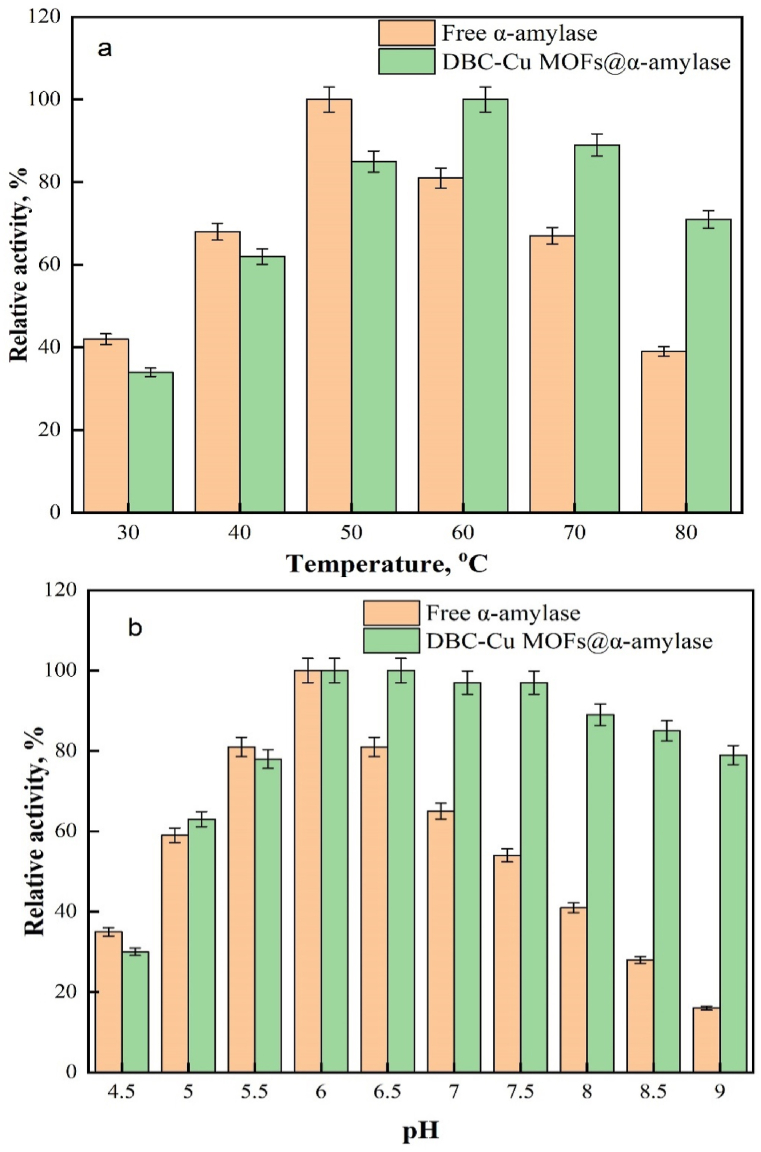
Effects of (a) temperature and (b) pH on α-amylase.

The optimal temperatures and pHs were determined for the free and immobilized α-amylases, since these are crucial in assessing their suitability for biotechnological processes. α-Amylases that function effectively at high temperatures hold significant potential for various industrial applications. In this study, the temperature supporting maximum activity of the free α-amylase was 50 °C, while the immobilized form exhibited an optimum temperature of 60 °C (Fig. 6a). The activity of the free enzyme was 81% at 60 °C. However, as the temperature increased, the enzyme activity gradually declined, reaching 39% at 80 °C. On the other hand, after immobilization, α-amylase demonstrated improved activity at elevated temperatures, including 71% at 80 °C. These results provided further evidence for the effectiveness of immobilization in preserving enzymatic activity at elevated temperatures and highlight the reduced sensitivity of immobilized α-amylases to temperature fluctuations. Similar findings have been reported in previous studies. For instance, when α-amylase from *Bacillus subtilis* was immobilized on a hydroxyapatite-decorated ZrO_2_ nanocomposite, it retained 80% of its activity after incubation at 80 °C [7]. In another study, α-amylase from Aspergillus oryzae was immobilized on a novel hybrid support and exhibited optimal activity at 60 °C [51]. Comparing the results of this study, the immobilized α-amylase demonstrated higher enzymatic activity at high temperatures (80 °C) than α-amylase from Arabian balsam immobilized on calcium alginate/Fe_2_O_3_ nanocomposite beads, which exhibited less than 55% activity at 80 °C [52]. The improved activity at elevated temperatures was attributed to alterations in the microenvironment surrounding the immobilized enzyme. This modified microenvironment shielded the enzyme from temperature-related fluctuations and protected it from thermal denaturation. As a result, the immobilized enzyme exhibited improved resistance to heat and maintained its activity at elevated temperatures [53]. These findings underscore the advantages of immobilization in increasing the stability and activity of α-amylase, particularly at elevated temperatures. The immobilization process provides a means to overcome the limitations associated with free enzymes and makes the immobilized α-amylase a valuable tool for various biotechnological applications that require efficient enzymatic activity at high temperatures.

The environmental pH influences the activity of α-amylase, so it was investigated in this study. The effects of pH on the catalytic properties of both the free and immobilized α-amylases were examined (Fig. 6b), which revealed that the catalytic activity of free α-amylase increased as the pH was increased from 4.5 to 6.0 and showed maximum activity at pH 6.0. However, as the pH increased from 6.0 to 9.0, the catalytic activity gradually decreased. These findings indicated that the optimal pH for free α-amylase is 6.0. In comparison, DBC-Cu MOFs@α-amylase resisted degradation by acidic or basic conditions and showed an expanded pH range. The catalytic activity of DBC-Cu MOFs@α-amylase increased as the pH was increased from 4.5 to 7.5. The maximum catalytic activity was observed within the pH range 6.0–7.5. However, above pH 7.5, the catalytic activity gradually decreased with further increases in the pH. These findings indicated that the DBC-Cu MOFs@α-amylase had a broader pH activity range, specifically within the pH range 6.0–7.5. The improved stability and pH resistance of the DBC-Cu MOFs@α-amylase was attributed to changes in the surface charges of the DBC-Cu MOFs and their impact on the ionic environment surrounding the active center of α-amylase. The immobilization of α-amylase within the DBC-Cu MOF structure provided protection and increased the stability of the enzyme. By modulating the surface charge, the DBC-Cu MOFs created a favorable environment for the immobilized α-amylase, which enabled it to withstand acidic and alkaline conditions more effectively. This modification of the enzyme microenvironment improved the resistance to pH changes, ultimately expanding its functional range and its applicability in diverse biotechnological processes. Previous studies investigated the immobilization of α-amylase with different materials and reported their pH profiles. For example, when α-amylase was immobilized on ultrafine polyvinyl alcohol, the optimal pH was 6.0, and the enzyme retained approximately 80% of its activity at pH 8.0 [49]. In another study, the α-amylase from *Bacillus subtilis* was immobilized on a metal-organic framework nanocomposite, and the optimal pH was 6.5, with the enzyme maintaining over 70% of its activity at pH 8.0 [32]. Acet et al. documented the immobilization of α-amylase onto poly(2-hydroxyethyl methacrylate) that attached to copper. The results of the study revealed that the immobilized enzyme exhibited enhanced stability within a pH range of 6–7.5 [54].

### 3.7 Kinetic behavior of a-amylases

The rates of the reaction were measured at different substrate concentrations in sodium acetate buffer (pH 5.5) with both free α-amylase and BDC-Cu MOFs@α-amylase. The double reciprocal method was used to plot the data and determine the values of Km and Vmax (Table 2). For free α-amylase, the linear regression equation was y = 1.644x + 19.18, with a Vmax of 0.61 U/mg of protein. In contrast, for BDC-Cu MOFs@α-amylase, the linear regression equation was y = 2.689x + 14.69, with a Vmax of 0.37 U/mg of protein. These Vmax values indicated that the structure of the enzyme was altered by covalent interactions with the cross-linker, which increased stability of the BDC-Cu MOFs@α-amylase. The Km value, which indicates the affinity between the enzyme and substrate, was significantly lower for BDC-Cu MOFs@α-amylase (5.46 mM) than for free α-amylase (11.67 mM).

**Table 2:** The kinetic behavior of free and immobilized α-amylase.

Parameter	Free α-amylase	BDC-Cu MOFs@α-amylase
Km (mM)	11.67	5.46
Vmax (U/mg.min)	0.61	0.37

This suggested that the enzyme immobilized on the surface of BDC-Cu MOFs had more accessible active sites and increased affinity of α-amylase for the starch substrate. The covalent interactions and immobilization of α-amylase onto the BDC-Cu MOF structure enhanced the stability and substrate affinity of the enzyme, making it a promising system for improved enzymatic activity in starch degradation. Atiroğlu et al. observed similar trends for the immobilization of α-amylase on a metal-organic framework. They found decreases in both the Km and Vmax values compared to those of the free α-amylase enzyme [32]. The decrease in Km indicated an increased affinity between the immobilized α-amylase and the substrate, while the reduction in Vmax suggested a decrease in the maximum reaction rate. These findings were consistent with the notion that enzyme immobilization can alter the enzyme structure and microenvironment and change the reaction kinetic. A study by Atiroğlu et al. provided further evidence that immobilization, such as with metal-organic frameworks, can impact the enzymatic activity and substrate affinity of α-amylase [32].

### 3.8 Enhancement of the antioxidant capacity of certain foods

Polyphenols are natural compounds found in various plant-based sources, such as cereals, vegetables, fruits, flowers, and tea [55]. They are a significant class of secondary plant metabolites [56]. Numerous studies have highlighted the positive health benefits of polyphenolic substances, including antioxidant, antiviral, anti-inflammatory, antithrombogenic, antiallergenic, antiasthma, antidiabetic, and anticancer properties [[57], [58], [59]]. One notable aspect of polyphenols is their association with dietary fiber. Prior research demonstrated that treating cereals with hemicellulose, a dietary fiber, increased the antioxidant activities of the cereals [60]. This suggested that the presence of hemicellulose, which is rich in polyphenols, could enhance the overall antioxidant potentials of cereals. Phenolic compounds are potent antioxidants found in various plant-based foods. Some of these compounds are bound to starch molecules, limiting their antioxidant activity. By breaking down the starch, α-amylase increased the accessibility of these antioxidants. In this study, both maize flour and finger millet samples were treated with free and immobilized α-amylase, and the polyphenol contents were measured before and after the enzyme treatment. The results are presented in Table 3. Compared to the control samples, the total phenolic content of the maize flour treated with free α-amylase was increased by a factor of 1.28, while the immobilized α-amylase treatment resulted in a 1.39-fold increase. Similarly, in the case of finger millet, the free α-amylase treatment led to a 1.35-fold increase in total phenolic content, and the immobilized α-amylase treatment resulted in a 1.64-fold increase. Interestingly, the use of a protease enzyme in a separate experiment also demonstrated increased antioxidant activity, possibly due to reduced interactions between the proteins and phenolic compounds [61]. The results highlighted a significant correlation between the total phenolic contents of maize flour and finger millet and the IC_50_ values obtained from antioxidant activity assays. As the phenolic content was increased, the IC_50_ values for the ABTS and DPPH assays decreased, indicating enhanced antioxidant activity. Additionally, when maize flour was treated with the immobilized enzyme, the IC_50_ values showed 1.44-fold (DPPH) and 1.53-fold (ABTS) increases. Similarly, the treatment of finger millet with immobilized α-amylase resulted in 1.33-fold (DPPH) and 1.26-fold (ABTS) increases in the IC_50_ values. These results demonstrated that treatment with α-amylase enhanced the antioxidant capacities of the maize flour and finger millet extracts, as evidenced by the decreased IC_50_ values obtained from the ABTS and DPPH assays.

**Table 3:** Improved antioxidant capacities of certain foods with free and immobilized α-amylases.

	Maize flour sample					Finger millet sample				
	Phenolic content (μg/g)	DPPH		ABTS		Phenolic content (μg/g)	DPPH		ABTS	
		IC_50_	R^2^	IC_50_	R^2^		IC_50_	R^2^	IC_50_	R^2^
Control food	18 ± 0.73	69 ± 0.17	0.995	13 ± 0.31	0.996	14 ± 0.42	52 ± 0.21	0.997	12 ± 0.07	0.992
Food treated with BDC-Cu MOFs@α-amylase	25 ± 0.55	48 ± 0.94	0.986	8.3 ± 0.94	0.992	23 ± 0.98	39 ± 0.53	0.989	9.5 ± 0.04	0.994
Food treated with Free α-amylase	23 ± 0.36	51 ± 0.85	0.981	9 ± 0.52	0.995	19 ± 0.61	41 ± 0.33	0.991	10 ± 0.12	0.988

Half-maximal inhibitory concentration (IC_50_); R-squared value (R^2^).

Treating certain foods with α-amylase increased their antioxidant capacities. α-Amylase specifically acts on starch and breaks it down into smaller molecules. Many antioxidant compounds in foods are bound to starch molecules, which limits their antioxidant activities. By breaking down the starch, α-amylase increases the accessibilities of these antioxidants [1]. In a related study conducted by Dey and Banerjee, α-amylase was used to increase the antioxidant capacity of wheat [62]. Similarly, Yu et al. utilized a combination of cellulase and α-amylase to release phenolic acids from barley, thereby enhancing its antioxidant characteristics [63].

## 4 Conclusion

In conclusion, copper-based metal-organic frameworks (MOFs) were synthesized with a casting approach and used to immobilize α-amylase. The immobilized α-amylase exhibited superior properties compared to the free form. Covalent binding of α-amylase to the BDC-Cu MOFs resulted in a high immobilization yield (81%) and activity yield (89%). Characterization techniques, including FTIR, SEM, EDX, and XRD, confirmed the compositions, morphologies, and crystalline characteristics of the synthesized materials. The zeta potential analyses provided insights into the surface charges of the BDC-Cu MOFs and BDC-Cu MOFs@α-amylase. The immobilized α-amylase maintained significant catalytic activity even after ten cycles, retaining 81% of its initial activity. Storage at 4 °C for eight weeks resulted in a higher activity retention rate for DBC-Cu MOFs@α-amylase (78%) compared to free α-amylase (49%). The immobilized form exhibited an optimum temperature of 60 °C, while the free form showed optimal activity at 50 °C. Both the free and immobilized α-amylase displayed peak catalytic activity at pH 6.0. The immobilized enzyme had a lower Km value (5.46 mM) and a comparable Vmax value (0.37 U/mg of protein) compared to the free enzyme (11.67 mM and 0.61 U/mg of protein, respectively). Furthermore, treatment of maize flour and finger millet samples with the free and immobilized α-amylase resulted in increased total phenolic content and enhanced antioxidant activity. These findings highlight the potential of BDC-Cu MOFs as effective supports for enzyme immobilization, offering improved enzymatic performance and potential applications in various industries. The utilization of BDC-Cu MOFs as a support material for enzyme immobilization opens up various applications in the food and biotechnology industries.

## 5 Materials and methodology

The following materials were procured from Sigma‒Aldrich Chemical Co. (St. Louis, MO): α-amylase from *Bacillus subtilis* (CAS Number: 9000-90-2; mol wt: 58–62 kDa), dimethylformamide (DMF), trimethylamine (TMA), starch, DPPH• (2,2- diphenyl-1-picrylhydrazyl), ABTS•+ (2,2- azino-bis(3-ethylbenzthiazoline-6-sulfonic acid), 1-ethyl-3-(3-dimethylaminopropyl) carbodiimide (EDC) (98%) N-hydroxysuccinimide (NHS) (95%), 1,4-benzene dicarboxylic (BDC, 98% from Merck) and copper nitrate Cu(NO_3_)_2_. Maize flour and finger millet samples were procured from a local market in Jeddah, Saudi Arabia.

### 5.1 Carrier preparation

To create the material support, a solution was prepared by dissolving 15 mM Cu(NO_3_)_2_ and 15 mM 1,4-benzene dicarboxylic in 80 mL of DMF. The solution was stirred gently, and 3 mL of trimethylamine was slowly added dropwise. The mixture was sealed and stirred at 80 °C for 2 h. The resulting BDC-Cu MOF crystals were separated by centrifugation, washed with DMF and dried.

### 5.2 Immobilization process

To immobilize α-amylase on the BDC-Cu MOFs, 200 mg of BDC-Cu MOFs was added to 10 mL of phosphate-buffered saline (50 mM PBS, pH 7.4), followed by the addition of 30 mg of EDC. The mixture was continuously stirred for 1 h at room temperature before adding 30 mg of NHS and stirring for an additional 1.5 h at room temperature. Next, a Falcon tube containing 80 units of α-amylase in 10 mL of PBS was used to immobilize the enzyme end-over-end for 12 h at room temperature. The resulting product (BDC-Cu MOFs@α-amylase) was separated by centrifugation and washed with phosphate-buffered saline, and the protein content was determined with the Bradford technique with bovine serum albumin as the reference standard [64]. The immobilization yield (IY%) and activity yield (AY%) were calculated with the following formulas:

$$\text{Immobilization}\;\text{Yield}\;\left(\text{IY}\%\right)=\frac{\;\text{Amount}\;\text{of}\;\text{protein}\;\text{introduced}\;-\;\text{Protein}\;\text{in}\;\text{the}\;\text{supernatant}}{\text{Amount}\;\text{of}\;\text{protein}\;\text{introduced}\;}\;X\;100$$

$$\text{Activity}\;\text{yield}\;\left(\text{AY}\%\right)=\frac{\text{Immobilized}\;\text{enzyme}\;\text{activity}}{\text{Iniatial}\;\text{activity}}\;X\;100$$

### 5.3 α-Amylase activity measurements

The Miller method was employed to determine the activities of both the immobilized and soluble forms of α-amylase [65]. To determine the activity of the immobilized enzyme, a standard protocol was followed with 10 mg of BDC-Cu MOFs@α-amylase. The immobilized and soluble forms of the enzyme were separately mixed with 1 mL of a 1% starch solution prepared in sodium acetate buffer (50 mM, pH 5.5) and incubated at 37 °C for 30 min. To develop the color, 1 mL of DNS reagent was added, but for the immobilized enzyme, it was first separated from the reaction and washed with distilled water before adding the DNS reagent. The reaction mixture was then incubated at 37 °C for 30 min, and the absorbance was measured at 560 nm.

### 5.4 Characterization of the material support

The morphologies of both BDC-Cu MOFs and BDC-Cu MOFs@α-amylase were analyzed with scanning electron microscopy coupled with energy dispersive X-ray spectroscopy (FEG-SEM: Quattro S FEG, SEM-Thermo Fisher, NL). Fourier transform infrared spectroscopy (FTIR, PerkinElmer Spectrum 100) was utilized to characterize the functional groups present in the BDC-Cu MOFs and BDC-Cu MOFs@α-amylase. An XRD system (XMD-300, UNISANTIS GERMANY, XQ Suite Software) was used to study the crystallite sizes and structural phases of the nanostructured BDC-Cu MOF samples. The zeta potential of the support was measured with a Malvern laser particle size analyzer (Zetasizer Ver. 7.12, UK).

### 5.5 Reusability and storage stability

Immobilized enzymes offer significant benefits over their free counterparts in terms of reusability. To investigate the reusability of the immobilized enzyme under optimal conditions, BDC-Cu MOFs@α-amylase was removed from the reaction mixture after the initial use by centrifugation, and any remaining substrate or product was washed away with sodium acetate buffer (50 mM, pH 5.5). These steps were repeated after each cycle, and a new substrate solution was added. The residual activity was then calculated as a percentage of the initial use (100%).

To evaluate the stability of free α-amylase and BDC-Cu MOFs@α-amylase during storage, both were kept at 4 °C, and their residual activities were measured on a regular basis over a period of eight weeks to determine if enzyme denaturation had occurred and affected their activities. The reported values represent the average of three measurements.

### 5.6 Effect of pH and temperature

The activities of free α-amylase and BDC-Cu MOFs@α-amylase were measured across different pH ranges (4.0–9.0) with various buffers: sodium acetate (pH 4.0–6.0), sodium phosphate (pH 6.5–7.5), and Tris-HCl (pH 8.0–9.0). The control value (100%) used in calculating the residual activity was based on activity at the optimum pH.

The activities of the free and BDC-Cu MOFs@α-amylase were evaluated in sodium acetate buffer (50 mM, pH 5.5) at various temperatures (30–80 °C) for a period of 15 min. The control value (100%) used in calculating the remaining percent activities of the free and immobilized enzymes was based on the activity at the optimal temperature.

### 5.7 Kinetic parameters

The maximum reaction velocities (Vmax) and Michaelis‒Menten affinity constants (Km) for free α-amylase and BDC-Cu MOFs@α-amylase were determined by measuring their activities with starch as the substrate according to the method of Choi et al. [66]. The Michaelis‒Menten equation was fitted to the experimental data with nonlinear regression analysis. The activity assay was conducted at pH 5.5 and 37 °C, with substrate concentrations ranging from 1.5 to 4 mg. The experiments were repeated three times, and the reported Vmax and Km values were the averages with standard errors.

### 5.8 Increased antioxidant capacities of certain foods treated with α-amylase

To conduct these experiments, maize flour and finger millet samples were ground into fine powders. Prior to testing, the powders were sieved through a 1 mm sieve. Each sample (0.5 g) was then combined with 4 mL of 0.1 M acetate buffer (pH 5.5) and autoclaved. After cooling, the autoclaved samples were treated with either free α-amylase (1 mL, 10 U) or BDC-Cu MOFs@α-amylase (16 mg, 10 U). The mixtures were incubated at 50 °C for 2 h [67]. To terminate the enzymatic reaction, the temperature of the mixture was raised to 95 °C for 3 min. For the control samples, 1 mL of acetate buffer was used instead of the enzyme. After 1 h of refluxing at 50 °C with 10 mL of distilled water, the reaction mixtures were obtained. To evaluate the degrees of hydrolysis, the total phenolic contents (TPCs) of the control samples and samples treated with the enzyme were determined with the methods described by Velioglu et al. [68]. The ABTS•+ and DPPH• scavenging activities of the samples were assessed with the techniques outlined by Ao et al. [69] and Re et al. [70], respectively.

## Ethics

Not applicable.

## Additional information

No additional information is available for this paper.

## Data availability statement

Data will be made available on request.

## CRediT authorship contribution statement

**Sami A. Al-Harbi:** Writing – review & editing, Methodology, Funding acquisition, Conceptualization. **Yaaser Q. Almulaiky:** Writing – original draft, Validation, Supervision, Resources, Methodology, Investigation, Formal analysis, Data curation.

## Declaration of competing interest

The authors declare the following financial interests/personal relationships which may be considered as potential competing interests: Sami A. Al-Harbi reports financial support was provided by 10.13039/501100011821Ministry of Education in Saudi Arabia. Sami A. Al-Harbi reports a relationship with Umm Al-Qura University that includes: employment.
