# Supplementary material for: Copper-based metal-organic frameworks (BDC-Cu MOFs) as supporters for α-amylase: Stability, reusability, and antioxidant potential
Source: Heliyon. 2024 Mar 19;10(6):e28396. doi: 10.1016/j.heliyon.2024.e28396 (PMC10979214; doi:10.1016/j.heliyon.2024.e28396)
Supplement: Multimedia component 1 [file mmc1.docx]

**Copper-based metal-organic frameworks (BDC-Cu MOFs) as supporters for α-amylase: Stability, reusability, and antioxidant potential**

Sami A. Al-Harbi ^a^ and Yaaser Q. Almulaiky ^b,c,⁎^

^a^Department of Chemistry, University College in Al-Jamoum, Umm Al-Qura University, Makkah, Saudi Arabia.

^b^Department of Chemistry, Collage of Science and Arts at Khulis, University of Jeddah, Jeddah, Saudi Arabia.

^c^Chemistry Department, Faculty of Applied Science, Taiz University, Taiz, Yemen

^⁎^Corresponding author

Dr. Yaaser Q. Almulaiky

Email: yaseralmoliki@hotmail.com


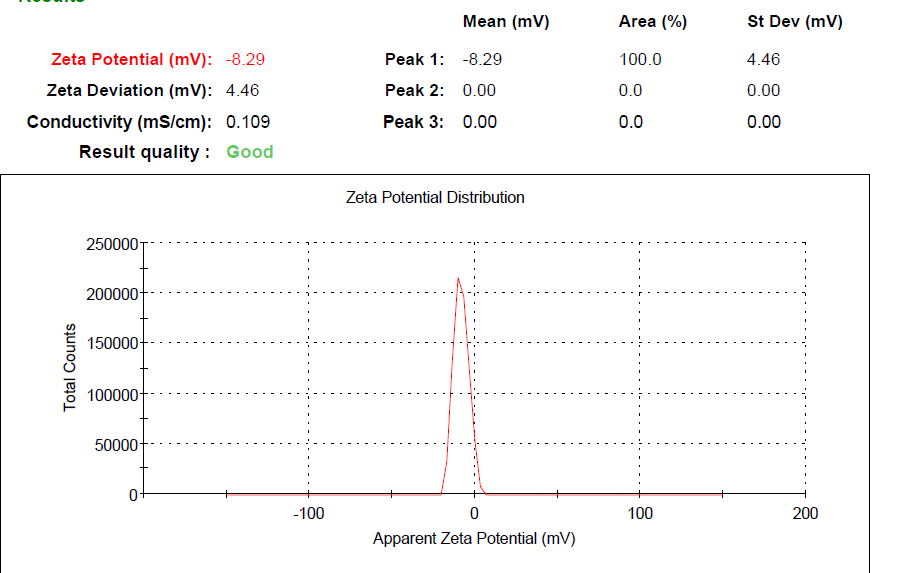


Figure 1S The zeta potentials of DBC-Cu MOFs


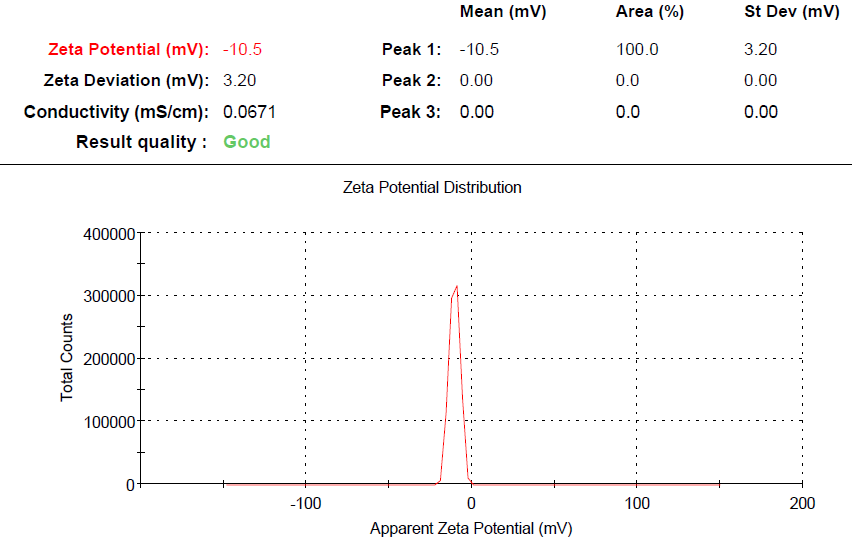


Figure 2S The zeta potentials of DBC-Cu MOFs@α-amylase
